# Illuminating gendered ageism with the WHO ageism experiences scale: a psychometric analysis and gender comparisons in a multi-country dataset

**DOI:** 10.1186/s12877-026-07898-6

**Published:** 2026-07-28

**Authors:** Xuefei Li, Tatiana Sorocan, Dina Ciubotaru, Diana Malasevschi, Viorica Zatic, Alla Constantinova, Marcela Bustamante, Nelson Andrés Tamayo, Deinedt Castellanos, Ahmed Alameen, Raja Alagaili, Jebril Gebril, Maya Abi Chahine, Aidan Timlin, Annie Wright, Aja Louise Murray

**Affiliations:** 1 Department of Psychology, University of Edinburgh, Edinburgh, UK; 2 HelpAge Moldova, Chisinau, Moldova; 3 HelpAge International, Latin America and the Caribbean Region, Colombia; 4 HelpAge International, London, UK; 5 Colombian Network for Dignified and Active Ageing (RedCoenve), Bogotá, Colombia; 6 Alsafwa Charity Organization, Tripoli, Libya; 7 University of Tripoli, Tripoli, Libya; 8 National Center for Disease Control, Tripoli, Libya; 9 University for Seniors Program, American University of Beirut, Beirut, Lebanon

**Keywords:** Gendered ageism, WHO Ageism Experiences Scale, Measurement invariance

## Abstract

Gendered ageism has been highlighted as an important global challenge, and progress in understanding and addressing it requires measures of ageism that are reliable, valid, and comparable across genders. However, there has been little examination of the extent to which existing measurement tools for ageism are appropriate for use within and for comparing ageism across genders. We used data collected across four diverse country contexts, Moldova, Colombia, Libya, and China (*N* = 1675), to examine the properties of the WHO Ageism Experiences Scale with respect to its capability for comparing ageism across genders and for illuminating selected aspects of gendered ageism — specifically, whether the scale functions equivalently across gender groups and whether men and women differ in their levels of ageism experiences and in the associations between ageism and health. Findings supported the gender measurement invariance of the scale across the reflective indicators. Results also indicated no significant differences in the levels of ageism experienced by women versus men in these contexts. However, the associations between ageism and health-related outcomes were significantly stronger for men than for women. The results supported the use of the WHO Ageism Experiences Scale across genders and highlighted a complex relationship between gender and ageism. These findings suggested that while experiences of ageism may not differ significantly between men and women, the consequences of ageism for health outcomes may vary, underlining the need for further research to better understand gendered dynamics in ageism.

Ageism can be defined as stereotypes, prejudice and discrimination based on age and can be observed in self-directed, interpersonal and institutional forms [1]. Stereotypes refer to cognitive aspects of ageism, prejudice to affective aspects, and discrimination to behavioural aspects. Examples of ageism might include being turned down for a job on the basis of age (discrimination); pitying someone based on their age (prejudice); or assuming that someone is not capable of autonomous decision-making due to their age (stereotyping) [2].

Ageism is relevant for all age groups but ageism against older adults has been the most extensively researched and has been associated with a wide range of negative outcomes [3–5]. For example, ageism has been linked to an increased prevalence of chronic health conditions [6, 7], higher risk of cognitive decline [8], worse mental health [9–11], has been found to create barriers to obtaining healthcare services [12] and even to affect healthy life expectancy [13].

In recognition of the consequent urgent need to tackle ageism, the World Health Organization (WHO) received a mandate from its member states in 2016 to lead a global campaign to combat ageism. As part of the campaign, a comprehensive evidence synthesis was conducted which summarised the current state of knowledge and provided recommendations for future research. One key recommendation was the need to explore how ageism intersects with other forms of social disadvantage — an idea grounded in the framework of *intersectionality*. Intersectionality highlights how multiple dimensions of identity, such as age, gender, and race, collectively shape individuals’ experiences of advantage or disadvantage [14]. Rather than considering each factor separately, this approach underscores that intersecting identities may produce compounded or unique forms of marginalisation, and may serve as potential social determinants of health disparities.

Social dominance theory [15] posited that both individual and structural forces contribute to group-based hierarchies and systems of oppression of privilege for dominant groups while marginalising others. It is widely acknowledged that, across societies, women and older individuals often occupy less dominant positions shaped by long-standing gendered and power relations [16, 17]. Gender and age function as interrelated rather than independent identity dimensions, collectively influencing one’s exposure to and experience of stereotypes, prejudice, and discrimination. More importantly, according to Rocha et al [18], such intersections encompass gender norms and roles that influence perceptions of ageing, not only gender/sex. For example, cultural understandings of ageing frequently intersect with gender ideals: ageing is often associated with decline, while femininity is sometimes linked to youth and beauty. These “double-standard” narratives contribute to women being judged more negatively for age-related changes than men, thereby reinforcing social inequities in respect, visibility, and opportunity.

From a policy and human rights perspective, the report A/76/157 by the Independent Expert on the enjoyment of all human rights by older persons, Claudia Mahler, highlighted the gendered effects of ageing, focusing on the key human rights challenges faced by older women. Although women constitute the majority of the older population globally, particularly among the oldest age groups, their specific needs and experiences often remain underrepresented. She further emphasised that strategies addressing gendered ageism should not apply a uniform approach to ‘older persons’ but must integrate a gender-sensitive perspective. Moreover, she urged all relevant stakeholders to strengthen attention to the intersection of ageing and gender in data collection, research, and analysis, and to enhance the visibility of older women within human rights frameworks and mechanisms.

This topic has also been attracting increasing attention within the academic community. Within the literature, *gendered ageism* has been identified as an area requiring special attention because of the potential compounded disadvantage conferred by the intersection of age and gender [1, 19, 20]. Gendered ageism refers to women and men experiencing ageism differently, with the “double jeopardy” of gender and age contributing to compounded challenges and heightened vulnerability for older women across various domains of social life [1, 21]. Some empirical evidence points to the nature of this double jeopardy. Firstly, older women may be particularly adversely affected by negative views of ageing and feel intensified pressure compared to men to hide visible signs of ageing. For example, according to Ishikawa [22], in comparison to men, women were more likely to view societal beliefs about ageing negatively and to fear their own ageing. Mackenstadt and Adams-Price [23] also found that negative ageing stereotypes, particularly fear of dependency, were associated with higher levels of depression and anxiety in women compared to men. Gendered ageism also manifests in the expectation that older women should conceal their age-related changes [24]. For example, evidence suggested that some older women make considerable efforts to manage their appearance, including using cosmetics and hair dye, to avoid being labelled as “old” [25, 26]. According to Cecil et al [27] older women engage in masquerades to conceal signs of ageing and preserve youthfulness in an effort to avoid ageism.

Older women also tend to be perceived as ‘old’ at a younger age. Evidence from the European Social Survey (ESS) round 9 (2018/19), as reported by Billari et al [28] showed that women across all countries become “old” before men, with such views largely driven by men. Further, gendered ageism is present in media representations, with older women receiving less visibility and more negative portrayals than older men. Drawing on data from three decades (1994, 2004, and 2014), [29] found that while both older men and women tend to become invisible in mainstream media, older women are disproportionately underrepresented compared to older men. Edström [30] found that older men on Hungarian evening news were more often portrayed as affluent, powerful, and active, while older women appeared in ordinary, family-oriented roles and less-productive activities. Similar trends were observed in the U.S., where older adults were underrepresented and typically depicted as young-old, male, white, middle-class, able-bodied [31].

In the labour market, older women are also often disadvantaged, with research suggesting that gendered discrimination resulted in women receiving 27% less annual pension payments than men in the OECD countries [20]. In the context of law and policy, [32] reported that age discrimination laws have been markedly less successful in improving labour market outcomes for older women relative to older men. Further, in healthcare settings, men have also been found to receive more aggressive treatment for age-related chronic illnesses, which women were perceived as too frail to undergo [33]. Additionally, according to UN Women [34], older women require more long-term care (LTC) compared to men, and also contribute more unpaid long-term care to their family members.

Yet, there remain many unanswered or only partially answered questions in relation to gendered ageism and compared to the overall literature on ageism, gendered ageism has received comparatively little attention. Our review of the relevant literature revealed a scarcity of quantitative studies examining gender differences in ageism; the existing evidence is predominantly qualitative or theoretical. This imbalance suggests a rich theoretical foundation that is not yet supported by robust empirical data. Addressing this gap requires reliable, valid, and comparable measures of ageism across genders. This is particularly important because, when researchers employ quantitative instruments to assess ageism, they must consider whether these tools function equivalently across gender groups. Gender may influence how individuals experience and perceive age-based discrimination, prejudice and stereotypes. Therefore, it is essential to develop and use instruments that demonstrate measurement equivalence across gender, ensuring that ageism is assessed consistently across both genders. Establishing gender scalar invariance of an instrument is particularly valuable, as it indicates that both factor loadings and item intercepts are equivalent across groups, thereby allowing for meaningful comparisons of mean levels of ageism between men and women.

Recently, the WHO Ageism Experiences Scale was developed to address a gap in measures of ageism that conformed to contemporary understandings of ageism and that can be used across the world. Whilst it was not explicitly designed to capture gendered ageism, its use to illuminate gendered ageism was implicit in its purpose as a tool that could be used across diverse groups and contexts. To help ensure relevance across genders, during its development, care was taken to include experts of different genders in the conceptualisation and item review phases (Murray & de la Fuente-Núñez [2]. Further, validation studies aimed to recruit samples with good representation of males and females. However, no study to date has explicitly evaluated the WHO Ageism Experiences Scale in terms of its capacity to elucidate ageism from a gender perspective. The goal of the present study was, therefore, to utilise available data on the WHO Ageism Experiences Scale to examine core psychometric properties with respect to gender, including gender measurement invariance. If gender invariance was supported, we subsequently examined whether older men and women differed in their experiences of ageism and explored whether gender moderated the relationship between ageism experiences and indicators of health and wellbeing. Overall, without measurement invariance, it remains unclear whether observed gender differences reflect true differences in the underlying construct or are the results of measurement error. Such situations may lead the scale to perform differently across gender groups, resulting in potential misinterpretation and invalid comparative conclusions.

It is important to note that gendered ageism, understood in its fullest intersectional sense, encompasses not only differences in the levels of ageism experienced by men and women, but also gendered dimensions of how ageism is socially constructed, internalised, as well as compounded by other axes of identity such as race, class, and sexuality [19]. From this perspective, the present study addresses a more bounded set of questions: whether the WHO Ageism Experiences Scale demonstrates gender measurement invariance, and whether gender moderates ageism levels and ageism-health associations. These questions are necessary preconditions for any broader intersectional empirical enquiry, but do not themselves constitute a comprehensive test of the intersectional theory. This study used data from four upper middle-income countries (UMICs) (Moldova, Colombia, Libya and China), in which we administered the WHO Ageism Experiences Scale as well as the same set of health and well-being outcomes. Using data from these contexts can help address the global evidence gap by capturing gendered ageism experiences in underrepresented settings, particularly given that most existing studies have been conducted in high-income countries.

## Methods

### Participants

Analyses were based on a combined dataset from Moldova, Colombia, Libya, and China, comprising a total of *N* = 1675 older adults aged 60 years and above. This study forms part of a broader programme of work aimed at validating the WHO Ageism Experiences Scale and examining ageism experiences of older adults in underrepresented global contexts. Much of the existing empirical research on ageing, particularly quantitative and psychometric studies, has been conducted in high-income countries, while an estimated 80% of the world’s older people (aged ≥ 60 years) will live in predominantly middle-income and other resource-constrained settings by 2030, resulting in a relative lack of evidence from these contexts [35]. Focusing on non-high-income settings therefore addresses an important gap in the literature and supports the development of measurement tools that are applicable across diverse socio-cultural and economic contexts. The selection of the four countries was based on the availability of established collaborations with local partner organisations and research teams, which enabled standardised translation procedures following the WHO Ageism Scale manual (i.e., TRAPD method), consistent data collection procedures, and contextual appropriateness of the materials. These countries also represent diverse geographic regions (Latin America, North Africa, East Asia, Eastern Europe) and cover major world languages including Spanish, Arabic, Chinese, Russian, thereby broadening the linguistic and cultural reach of the research and strengthening the cross-contextual relevance of the findings.

Data were collected between June 2024 and May 2025. In each country, recruitment and fieldwork were coordinated by local academic partners and trained enumerators under the supervision of the University of Edinburgh. Before data collection began, all field teams participated in a comprehensive online training programme delivered by the University of Edinburgh. The training familiarised data collectors with the study objectives, survey materials, and ethical principles, and provided detailed instructions on participant approach, interview procedures, confidentiality, and handling of potentially sensitive questions. Mock interviews and role-play sessions were included to ensure standardisation across sites.

In Moldova and Libya, data collection was implemented by local survey agencies, whereas in Colombia, enumerators from four collaborating universities carried out the fieldwork. In China, trained university students from a partner institution carried out data collection. A pilot study involving 5–10 older adults in each country was conducted prior to the main data collection to identify potentially sensitive or ambiguous items and to refine culturally appropriate wording.

Participants were recruited through visiting spaces around community centres, health clinics, senior clubs, and public spaces commonly visited by older adults. All participants provided informed consent prior to taking part in the study. Participation was entirely voluntary, and participants were informed of their right to withdraw at any time without penalty. Eligibility criteria were being aged 60 years or older and capable of providing informed consent. Informed consent was obtained either verbally or in writing, depending on literacy levels and local ethical guidance. Surveys were administered using Computer-Assisted Personal Interviewing (CAPI) or paper–pencil versions, according to local feasibility and participant preference. Ethical approval for all four studies was obtained from the University of Edinburgh PPLS Research Ethics Committees (PPLSREC), with ref no. 310–2425/2 for Moldova, 27–2425/2 for Colombia, 241–2425/2 for Libya, and 39–2425/2 for China.

### Translation of the survey materials

We administered the survey in local languages (i.e., Romanian and Russian in Moldova, Spanish in Colombia, Arabic in Libya, and Simplified Chinese in China). All four countries employed the same translation procedure, following the Translation, Review, Adjudication, Pretesting, and Documentation (TRAPD) method as recommended in the official WHO Ageism Scale guidelines [36]. As a gold standard method, the TRAPD approach and its adaptations (e.g., translation by a single human translator and by machine translation, each conducted independently) have been shown in some studies to enhance translation quality and ensure data comparability across different linguistic and cultural groups [37, 38].

### Measures

#### WHO Ageism experiences scale

Ageism experiences of older adults were measured using the 15-item WHO Ageism Experiences Scale, which captures three domains—stereotypes, prejudice, and discrimination—across self-directed, interpersonal, and institutional levels. Participants rated items on a 5-point Likert scale ranging from “strongly agree” to “strongly disagree,” with a “don’t know/not applicable” option included. Three items were reverse scored so that higher scores suggested greater ageism experiences. Translated versions of the scale are accessible via the official WHO Ageism Scale website (https://www.aworld4allages.org/who-ageism-scale).

#### Health-related Quality of Life (HRQoL)

The 12-item Short Form Health Survey version 2 (SF-12v2) was used as an instrument for assessing health-related quality of life (HRQoL). It provides a concise yet comprehensive evaluation of physical and mental health via two composite scores: the Physical Component (PCS) and the Mental (psychological) Component (MCS) across diverse populations and conditions. The SF-12v2 has shown good reliability and validity across a range of settings, supporting its application in both clinical and research contexts, including use with older adult populations (e.g., [39, 40].

#### Well-being

Subjective well-being was assessed using the WHO-5 Well-Being Index, a brief and widely used measure designed to evaluate positive mood, vitality, and general interest in life. The scale consists of five items rated on a 6-point Likert scale ranging from “At no time” to “All of the time.” Its simplicity and efficiency make it suitable for use in diverse settings. The WHO-5 has demonstrated robust validity and reliability across various populations and has been validated in multiple languages and cultural contexts [41].

#### Loneliness

Loneliness among older adults was assessed using the UCLA Loneliness Scale, a widely recognised instrument originally comprising 20 items. The 6-item version (ULS-6) was employed to provide a brief yet psychometrically sound measure, validated for use with older populations [42]. Participants rated six items on a 4-point Likert scale (1 = “never” to 4 = “often”), with higher scores indicating greater levels of loneliness.

#### Demographic variables

Participants reported their *chronological age* in years as a continuous variable. *Gender/Sex* was self-reported by participants and coded as 0 = male and 1 = female. As the survey item assessed *sex* rather than gender identity directly, we use the term gender in the narrative, in line with the study’s conceptual framing of gendered ageism, and treat self-reported sex as a proxy for gender; in the tables and figures, we retain sex (male/female) to reflect the survey item as actually measured. Participants who preferred not to disclose their sex or provided non-binary responses were excluded from these comparisons due to small sample sizes. *Subjective social status* was measured using the MacArthur Scale of Subjective Social Status, on which participants placed themselves on a 10-rung ladder (1 = lowest, 10 = highest) representing their perceived standing in society relative to others, with higher scores indicating higher subjective social status. Participants’ *highest completed education level* was recorded using each country’s local education categories. To ensure cross-country comparability, responses were harmonised into a three-level variable representing low, medium, and high education. Specifically, “low education” referred to primary education or below, “medium education” included secondary or vocational education, and “high education” included college or university and above. A detailed mapping of this harmonisation across countries is provided in Appendix Table A1.

**Table 1:** Sample characteristics

	Overall *n* = 1675	Moldova *n* = 425	Colombia *n* = 398	Libya *n* = 350	China *n* = 502
Overall ageism experience	2.61 (0.60)	2.35 (0.59)	2.48 (0.62)	2.77 (0.53)	2.84 (0.50)
Self-directed ageism	2.55 (0.72)	2.38 (0.71)	2.28 (0.73)	2.72 (0.69)	2.78 (0.63)
Interpersonal ageism	2.5 (0.76)	2.17 (0.72)	2.35 (0.83)	2.63 (0.67)	2.79 (0.63)
Institutional ageism	3.02 (0.77)	2.76 (0.78)	3.1 (0.92)	3.2 (0.70)	3.05 (0.63)
Age	69.25 (7.21)	69.13 (6.79)	72.5 (7.91)	68.15 (5.89)	67.55 (6.98)
Number of women participants	939 (56.1%)	315 (74.1%)	301 (76%)	98 (28%)	225 (44.8%)
Subjective social status	4.39 (1.93)	4.79 (1.77)	3.22 (2.09)	5.39 (1.44)	4.28 (1.72)
Education (harmonised level)	1.77 (0.64)	2.29 (0.49)	1.41 (0.57)	1.76 (0.43)	1.61 (0.61)

### Statistical procedure

All analyses were conducted using R version 4.4.1. Descriptive statistics were first obtained to summarise sample characteristics and key study variables. Gender differences at item level and internal consistency for men and women were also examined to provide preliminary evidence. A confirmatory factor analysis (CFA) was then conducted on the self-directed ageism items using the *lavaan* package [43] to test their hypothesised unidimensional factor structure. It is important to note that only the self-directed ageism items were subjected to confirmatory factor analysis (CFA) and measurement invariance testing, as these items serve as reflective indicators representing an underlying latent trait of internalised ageism, while the interpersonal and institutional ageism items are formative indicators that capture the heterogeneous experiences across various contexts and thus are unsuitable for factor analysis [44]. In addition, to account for contextual variability across settings, country was included as a covariate in the CFA models, which allow country to predict each item directly. Model fit was evaluated with the Comparative Fit Index (CFI) and Standardized Root Mean Square Residual (SRMR), using thresholds of CFI > 0.90 and SRMR < 0.08 to indicate acceptable fit [45, 46].

Then, measurement invariance across gender group was examined through multi-group CFA (MGCFA) by sequentially testing configural, metric, and scalar models. Country was again included as a covariate in these models to control for potential country-specific effects on items. Each successive model was compared to the previous one using chi-square different test and by inspecting changes in fit indices. Specifically, given the sensitivity of the chi-square difference test to large sample sizes, measurement invariance was evaluated primarily on the basis of changes in fit indices: invariance was considered supported when changes in CFI and SRMR did not exceed 0.02 and 0.03, respectively, with the chi-square difference test treated only as a supplementary reference, in line with recommendations for large-sample contexts [47, 48].

Finally, if measurement invariance was supported, multilevel linear mixed-effects models (LMMs) were employed to examine gender differences in overall and domain-specific ageism experiences, as well as their associations with health and well-being outcomes. Although scalar invariance permits the comparison of latent means within the CFA framework, we did not estimate latent mean differences using MGCFA. Instead, we examined gender differences using LMMs based on observed composite scores. This approach was chosen to allow for the modelling of associations between ageism and health outcomes while accounting for the nested structure of the data (participants within countries). In addition, our analyses included domain-specific ageism (interpersonal and institutional), which are conceptualised as formative indicators and therefore not suitable for latent variable modelling within CFA. This approach also reflects a widely used analytical strategy in fields such as epidemiology and population health, as LMMs allow researchers to simultaneously examine group differences and their associations with outcomes while accounting for complex, multi-level data structures. All models accounted for the nested structure of the data, with participants clustered within countries, by including random intercepts for country. Specifically, for each outcome, a series of three hierarchical models was estimated. The first model (Model 0) served as a baseline model, including only a random intercept for country to account for clustering within sites. The second model (Model 1) added gender as a fixed effect to test for overall gender differences in ageism experiences. The third model (Model 2) further adjusted for key covariates (i.e., age, education, and subjective social status) to determine whether any observed gender effects persisted after accounting for these factors.

The same analytical approach was then applied to assess gender differences in health outcomes, including physical and psychological health-related quality of life (HRQoL), subjective well-being, and loneliness.

## Results

### Descriptive statistics

#### Sample characteristics

Descriptive statistics of four samples were presented in Table 1. The mean age of the overall sample was 69.25 years, with participants in Colombia being slightly older and those in China slightly younger. Female participants accounted for 56.1% of the sample. Most participants positioned themselves at the midpoint of the social status scale, with Colombian participants reporting an overall lower subjective social status. Older adults in our sample, on average, experienced a moderate level of ageism, with institutional ageism emerging as the most prominent. Participants from Libya consistently reported the highest scores across all ageism dimensions.

#### Item-level gender differences in self-directed ageism

Table 2 presents item-level self-directed ageism experiences by gender. Overall, older men reported higher levels of ageism than older women. Men scored higher on all items, including stereotype (thoughts), prejudice (emotional) and discrimination (behavioural) items.

**Table 2:** Item level self-directed ageism experiences across gender

	Mean (SD)		N (%) of missing	
	Male	Female	Male	Female
Life purpose	2.70 (1.19)	2.28 (1.18)	38 (5.2%)	31 (3.3%)
Age burden	2.31 (1.04)	2.30 (1.18)	14 (1.9%)	28 (3%)
Age embarrasses	2.14 (1.06)	2.11 (1.11)	11 (1.5%)	19 (2%)
Discussion limit	2.81 (1.19)	2.66 (1.23)	17 (2.3%)	22 (2.3%)
Inappropriate limit	3.17 (1.1)	3.12 (1.21)	36 (4.9%)	29 (3.1%)

### Confirmatory factor analysis of the WHO ageism experiences scale (self-directed items)

According to Murray and colleagues [49], a one-factor model is preferred for the self-directed items of the WHO Ageism Experiences Scale. To verify whether this unidimensional structure was applicable to our combined sample, a univariate confirmatory factor analysis (CFA) was conducted on the five self-directed items. The results supported a unidimensional model across the overall sample, indicated by satisfactory fit indices before (CFI = 0.962, SRMR = 0.028) and after (CFI = 0.964, SRMR = 0.029) adjusting for country. Consequently, the one-factor model was established as the basis for examining the subsequent measurement invariance across gender.

We also assessed McDonald’s omega as an indicator of the internal consistency of the self-directed items. The results indicated acceptable internal consistency, with omega values of 0.62 for the full sample, 0.63 for males, and 0.62 for females.

### Measurement invariance across gender

We first assessed whether the one-factor model demonstrated acceptable fit separately for older men and women. The model fit was satisfactory for both men (CFI = 0.959, SRMR = 0.035) and women (CFI = 0.964, SRMR = 0.030). Consequently, data from both genders were combined to perform a multi-group confirmatory factor analysis (MGCFA), followed by tests of metric and scalar measurement invariance. Results were presented in Table 3. In summary, all tested models demonstrated good model fits. Regarding model comparison, the configural model showed good fit, suggesting that the same factor structure was applicable to both men and women. When factor loadings were constrained (metric invariance) and subsequently when intercepts were constrained (scalar invariance), the changes in fit indices remained within the recommended thresholds suggesting that both metric and scalar invariance were supported. Although the chi-square difference tests were significant, this was expected given their sensitivity to large sample sizes. Overall, these results provided evidence for measurement equivalence across gender, allowing meaningful comparisons of latent means between men and women.

**Table 3:** Fit indices and model comparison for measurement invariance models across gender

	χ^2^	DF	CFI	SRMR	∆CFI	∆SRMR	∆χ^2^	Pr(> χ^2^)
Configural	47.248	10	.962	.028				
Metric	51.194	14	.962	.032	0.000	0.004	3.946	0.413
Scalar	81.489	18	.945	.042	0.017	0.010	30.295	< .001

### Gender difference on self-directed ageism experiences

Given that scalar invariance was confirmed, we subsequently examined gender differences in ageism experiences using multilevel linear mixed-effects models. A preliminary unconditional model (Model 0) was estimated to assess the proportion of variance in ageism experiences attributable to between-country differences. The intraclass correlation coefficients (ICCs) for the overall and domain-specific ageism scores all greater than 0.10, which suggested substantial clustering by country and confirms the necessity of adopting a multilevel modelling approach. Consequently, all subsequent models incorporated random intercepts for country to appropriately account for contextual variability across sites.

As shown in Table 4, results suggested no significant gender differences in any of the ageism dimensions when gender was entered as the sole predictor. After additionally adjusting for age, education, and subjective social status, the gender effects remained non-significant. These findings suggested that experiences of ageism are largely comparable between older men and women across domains. In contrast, higher age and lower subjective social status were consistently associated with significant higher level of reported ageism, while higher educational attainment was linked to significant lower levels of self-directed and interpersonal ageism.

**Table 4:** Gender differences on ageism experiences

	*B* (SE)							
	Overall ageism		Self-directed ageism		Interpersonal ageism		Institutional ageism	
	Model 1	Model 2	Model 1	Model 2	Model 1	Model 2	Model 1	Model 2
Sex (Female)	0.001 (0.030)	0.002 (0.029)	0.031 (0.037)	0.033 (0.036)	−0.004 (0.038)	−0.008 (0.038)	−0.029 (0.040)	−0.023 (0.040)
Age		0.008 (0.002)***		0.011 (0.002)***		0.008 (0.003)**		0.001 (0.003)
Education (Medium)		−0.096 (0.034)**		−0.087 (0.044)*		−0.150 (0.045)**		−0.027 (0.049)
Education (High)		−0.161 (0.055)**		−0.240 (0.068)***		−0.224 (0.073)**		0.049 (0.078)
Social status		−0.065 (0.008)***		−0.068 (0.010)***		−0.066 (0.010)***		−0.067 (0.011)***

Model 1 includes gender as a fixed effect. Model 2 additionally adjusts for age, education (reference level: low), and subjective social status. All models include a random intercept for country

^*^p < .05, **p < .01, ***p < .001

### Gendered ageism and its impact on health and well-being outcomes

Finally, we examined whether the associations between ageism experiences and health and well-being outcomes differed by gender using multilevel linear mixed-effects models. This approach included a random intercept for country, thereby controlling for cross-national differences in health outcomes. The fixed effects included overall ageism, gender, and their interaction term (Ageism × Gender) to test whether the strength or direction of the association between ageism and health varied across genders. Model 1 included gender, ageism, and their interaction as fixed effects, while Model 2 additionally controlled for age, education, and subjective social status.

**Fig. 1:**
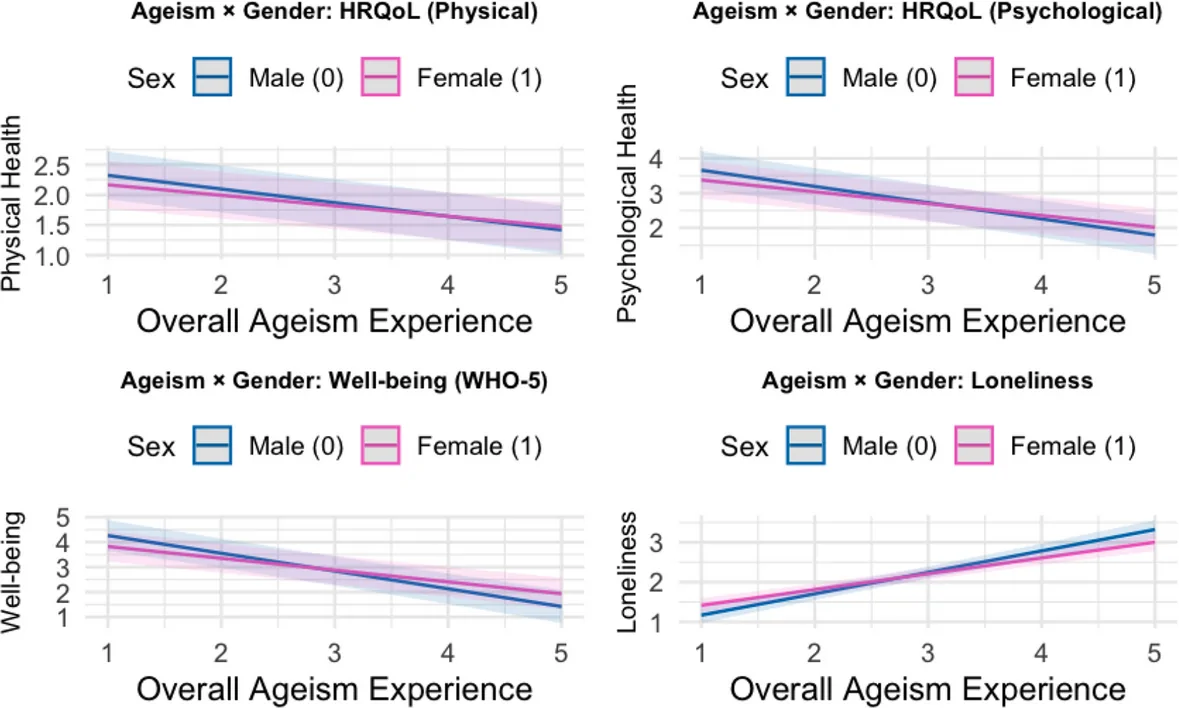
Moderating role of gender in the association between ageism and health

As shown in Table 5, ageism was significantly associated with poorer outcomes across all four health indicators: physical and psychological health-related quality of life (HRQoL), well-being, and loneliness. Gender differences were also significant, with older women generally reporting worse health outcomes than men across all health domains, including lower health-related quality of life, poorer well-being, and higher levels of loneliness. Notably, the interaction terms between ageism and gender were statistically significant for all outcomes except physical HRQoL, which suggested that the health effects of perceived ageism vary by gender. Specifically, the positive interaction coefficients for physical and psychological HRQoL and well-being, and the negative coefficient for loneliness (where higher scores indicate worse health), suggested that the detrimental impact of ageism was less pronounced among older women compared to men. In other words, older men appeared more vulnerable to the negative health consequences of ageism. The moderating role of gender in the association between ageism and health outcomes were also presented in Fig. 1.

**Table 5:** Interaction effect of gender and overall ageism on health and well-being

Parameter	HRQoL (physical)		HRQoL (psychological)		Well-being		Loneliness	
	Model 1	Model 2	Model 1	Model 2	Model 1	Model 2	Model 1	Model 2
Ageism	−0.271 (0.029)***	−0.228 (0.028)***	−0.510 (0.044) ***	−0.468 (0.044)***	−0.744 (0.071)***	−0.711 (0.071)***	0.556 (0.042)***	0.538 (0.042)***
Sex (Female)	−0.195 (0.099)*	−0.210 (0.096)*	−0.370 (0.152)*	−0.414 (0.151)**	−0.617 (0.244)*	−0.679 (0.243)**	0.349 (0.146)*	0.387 (0.145)**
Ageism × Sex	0.046 (0.036)	0.053 (0.035)	0.112 (0.056)*	0.128 (0.056)*	0.216 (0.090)*	0.239 (0.090)**	−0.125 (0.054)*	−0.141 (0.053)**
Age		−0.011 (0.002)***		−0.005 (0.002)*		−0.001 (0.004)		0.001 (0.002)
Education (Medium)		0.102 (0.027)***		0.054 (0.042)		0.066 (0.071)		−0.085 (0.041)*
Education (High)		0.154 (0.046)**		0.102 (0.067)		0.014 (0.109)		0.018 (0.064)
Social status		0.025 (0.006)***		0.049 (0.010)***		0.059 (0.016)***		−0.033 (0.009)***

Model 1 includes sex, ageism, and their interaction as fixed effect. Model 2 additionally adjusts for age, education (reference level: low), and subjective social status. All models include a random intercept for country

^*^p < .05, **p < .01, ***p < .001

## Discussion

The present study examined the WHO Ageism Experiences Scale from the perspective of its capability for comparing ageism across genders and illuminating selected aspects of gendered ageism in a combined sample of older adults from four UMICs — Moldova, Colombia, Libya, and China. Three main findings emerged from the analysis. First, scalar measurement invariance of the self-directed ageism items was supported across gender, indicating that the scale measures the underlying construct equivalently for older men and women and that gender comparisons of self-directed ageism scores are psychometrically justified. Second, no significant mean-level gender differences were found across any ageism dimension, suggesting that overall exposure to ageism is broadly comparable between older men and women in these contexts. Third, despite comparable mean levels, gender significantly moderated the associations between ageism and health outcomes, with the detrimental effects of ageism on physical and psychological health-related quality of life, wellbeing, and loneliness being stronger for older men than for older women.

Importantly, the support of scalar invariance suggested that the latent construct of self-directed ageism is conceptualised similarly among older men and women. By establishing scalar invariance, this study provided a robust foundation for comparing levels of internalised ageism across gender, helping ensure that observed similarities or differences reflect true variation in the underlying construct rather than measurement bias. Accordingly, this strengthens the conclusion that any observed gender differences in self-directed ageism can be interpreted as substantive rather than methodological. To our knowledge, this study is the first to establish measurement invariance of the WHO Ageism Experiences Scale across genders, contributing to the limited quantitative research on ageism experiences in diverse international contexts. Given that ageism is increasingly recognised as a critical global issue [50], and considering the compounded vulnerabilities introduced by gendered ageism, establishing reliable and gender-equivalent measurement instruments is essential. Thus, this study can serve as a starting point and provide a psychometric foundation for future research to explore gender differences in ageism rigorously and systematically across diverse cultural contexts. It is worth noting that the gender invariance confirmed in this study is based on a sample of older adults. Since ageism exists across all age groups (de la Fuente-Núñez et al. [51], examining measurement invariance across gender in younger populations with the scale is an important future direction.

Regarding gender differences in ageism experiences, no significant differences were found across any ageism dimension. Descriptively, women tended to report slightly higher overall and self-directed ageism, whereas men reported slightly higher interpersonal and institutional ageism. This pattern adds nuance to existing evidence: Allen et al [52] already noted that across the majority of studies reviewed, no significant gender differences in ageism levels were found, consistent with our results. One possible explanation is that women who have experienced lifelong sexism may become more habituated to discrimination, or may attribute certain experiences to gender rather than age, contributing to comparable or lower reported ageism levels [52]. The descriptive pattern that women score slightly higher on self-directed ageism is consistent with evidence that women show higher levels of internalised negative ageing stereotypes and ageing anxiety compared to men [53]. Interestingly, Lytle et al [53] also found that prior experience of stigma-based discrimination based on sexual orientation may foster coping resources that buffer the impact of subsequent age-based discrimination, thereby providing indirect evidence that women’s lifelong navigation of gender-based disadvantage may, paradoxically, equip them with greater resilience when confronting age-based discrimination in later life. At the same time, the pattern that men descriptively report slightly higher interpersonal and institutional ageism may reflect a more abrupt and novel confrontation with ageism in later life. Research suggested that gender substantially moderates how older adults are perceived by others: whereas women tend to be stereotyped as relatively incompetent regardless of age, older men experience a more dramatic shift — from being viewed as capable and self-sufficient in younger adulthood to being perceived as dependent in later life, with their competence ratings declining more sharply than women’s when they are seen to require assistance [54]. This is further supported by Clarke and Korotchenko [55], who argued that men’s sense of immunity to ageism in earlier adulthood is largely sustained by gender privilege and social status, but that this immunity is temporary. As physical decline becomes inevitable, men ultimately face a more disruptive confrontation with social exclusion than women, who have long navigated lower social expectations. This more identity-disrupting experience of ageism, especially for men who have benefited from age and gender privilege earlier in life, may also help explain the stronger associations between ageism and health outcomes observed among men in the present study.

It is also worth noting that, as reviewed in the introduction, women's experiences of ageism might be qualitatively different from men’s: women are more likely to fear their own ageing and view societal beliefs about ageing negatively [22], face greater pressure to conceal signs of ageing through appearance management [25–27], and are disproportionately underrepresented in media [29, 30]. This suggests that the forms and contexts of gendered ageism differ substantially between men and women even when mean levels are comparable, and that comparing mean scores alone is insufficient to capture the full complexity of gendered ageism experiences. Taken together, both older men and women are vulnerable to ageism, and gendered ageism may manifest *differently* rather than being necessarily more pronounced for one gender. The difference may yield equivalent overall self-reported ageism scores, while the nature and form of ageism experiences vary by gender. Importantly, while our measurement invariance testing suggested statistical equivalence of the ageism construct across gender groups, this does not imply that the experiences themselves are perceived or internalised in the same way. Invariance ensures that the scale functions similarly across groups, but it cannot capture potential qualitative differences in how individuals interpret, react to, or cope with ageism. In addition, the absence of a significant mean-level effect may be attributable to limited statistical power, coarse measurement granularity, or the likelihood that gendered ageism takes qualitatively different forms that are not adequately captured by mean-level comparisons. Thus, the absence of a statistical difference should not be interpreted as evidence against gendered experiences, but rather emphasises the need for more nuanced, item-level or qualitative investigations of how gender shapes ageism experiences.

Regarding the health impacts of ageism, both men and women experienced significant negative associations between ageism and physical, psychological, and wellbeing outcomes, but the detrimental effects appeared stronger among older men. This stronger association between ageism and health outcomes for men is supported by previous empirical research [10], which found that experiences of ageism had a greater impact on depression among men than women. Beyond the identity-disrupting nature of ageism for men discussed above, which may help to explain this association, another explanation is that men may have fewer psychosocial coping resources and less extensive social support networks to buffer the impact of perceived discrimination. This interpretation is consistent with prior evidence that older men are more vulnerable to stress-related health risks [56]. However, although prior research has demonstrated the buffering role of supportive social networks and coping strategies in mitigating the effects of ageism (e.g., [57], as well as gender differences in these resources (e.g., [58, 59], there is currently a lack of direct empirical evidence examining whether gender differences in social networks and coping strategies influence vulnerability to the health impacts of ageism. This represents an important direction for future research. In sum, this finding suggests that although exposure to ageism is widespread and its detrimental effects on health are significant, these health implications are not uniform and may depend on gendered pathways through which individuals internalise and respond to such experiences. Understanding these gender-specific mechanisms is essential for designing targeted interventions that address both the forms and the impacts of ageism across genders.

In summary, our study was the first to establish the measurement invariance of the self-directed items of the WHO Ageism Experiences Scale across gender in four culturally diverse countries. This provides a valuable psychometric foundation for gender comparisons of ageism experiences in future research. By confirming that the scale functions equivalently for older men and women, we offer a baseline for accurately capturing and interpreting gendered experiences of self-directed ageism in global ageing research.

Our findings carry several theoretical and practical implications. Theoretically, the results contribute to the growing discourse on gendered ageism, underscoring the importance of considering both common and distinct pathways through which men and women experience ageism. Practically, the availability of a gender-invariant tool allows researchers, practitioners, and policymakers to use the WHO Ageism Experiences Scale with greater confidence when designing, monitoring, and evaluating interventions aimed at reducing ageism among older adults. Moreover, this work advances the cross-cultural validation of the scale, which is essential for global health and ageing and gender equity initiatives.

Despite these contributions, several limitations of the present study warrant consideration. One concern that should be noted is the conceptual scope of the measure used. Although the WHO Ageism Experiences Scale was developed to be applicable across diverse populations, it was not specifically designed to capture the intersection of ageism and sexism. As a result, the present study is able to illuminate certain aspects of gendered ageism — notably, whether men and women experience different levels of ageism and whether gender shapes the consequences of ageism for health — but cannot directly assess the compounded or synergistic disadvantage that intersectional theories of gendered ageism posit. Future research might consider adapting or extending the scale to incorporate gendered dimensions more explicitly. For example, rephrasing items to reflect combined attributions or developing parallel forms of scales with ageism and sexism items could help assess the intersectionality of these constructs. This would be especially useful for capturing experiences of individuals who may face compounded disadvantages. Second, while the inclusion of diverse country contexts enhances the generalisability of findings, it also introduces heterogeneity in socio-cultural norms, gender roles, and policy environments that may influence how ageism is perceived and reported. All four countries included in this study are UMICs, and the results should therefore be interpreted as evidence about gendered ageism in these specific diverse settings rather than as globally generalisable conclusions. The inclusion of only one widely studied ageing research context (i.e., China) means that it is more difficult to interpret the findings in terms of existing ageism knowledge, largely drawn from high-income country settings (e.g., USA, Canada, UK, or Germany) where much of the existing ageism literature has been generated and where institutional, cultural, and policy environments differ substantially. Future research should extend these analyses to additional contexts, including high-income countries, to further establish the capability of WHO Ageism Experiences Scale to capture and illuminate gendered patterns of ageism experiences. Furthermore, although random intercept for country was included in the multilevel models to account for between-country differences, this approach does not fully capture how cultural norms and structural gender inequalities may shape ageism experiences across contexts. Gender roles, labour market participation, and social expectations on gender differ substantially across countries, which may influence both the manifestation of ageism and its consequences for men and women. As such, the observed gender patterns should be interpreted within the socio-cultural contexts represented in this study. Specifically, the extent to which similar patterns would be observed in high-income countries where gender inequalities may differ in magnitude and form remains an open question. Prior research has shown that macro-level sociopolitical factors such as economic prosperity buffer against the negative impact of ageism [60]. Future research should examine these dynamics across a broader range of contexts and explore potential interactions between gender and country-level factors. Finally, the cross-sectional nature of the data limits our ability to obtain temporal dynamics or life-course trajectories of gendered ageism. Longitudinal studies are needed to understand how internalised age stereotypes evolve and how cumulative or changing exposure to ageism differentially affects men and women over time. Such studies could illuminate the developmental mechanisms underpinning the stereotype embodiment theory and offer a more nuanced understanding of gendered vulnerabilities.

In conclusion, the present study offers a contribution to the literature on ageism by providing empirical evidence for the gender measurement invariance of the WHO Ageism Experiences Scale in older adults across four countries. While average scores may not differ significantly by gender, older men and women seem to experience ageism in different ways. Future research should further unpack these gendered patterns through longitudinal, qualitative, and intersectional approaches to inform more inclusive and effective policies and interventions. Ultimately, improving the measurement of gendered ageism is not only a methodological task but also a crucial step toward recognising and addressing the intersectional inequalities that shape how older adults experience ageing.

## Data Availability

Dataset of current study are available from the corresponding author upon reasonable request.

## Appendix 1

### Harmonisation of education variable across sites

**Table:**

Country	Original Category / Label	Harmonised Category
**Moldova**	Less than primary education or Primary school	Low
	Lower secondary school	Medium
	Upper secondary school	Medium
	Vocational education / Post-secondary non-tertiary education	Medium
	Short-cycle tertiary education	Medium
	Bachelor’s or equivalent level	High
	Master’s or Doctoral equivalent level	High
	Other	Excluded (set to NA)
**Colombia**	Primaria incompleta	Low
	Primaria completa	Low
	Bachillerato incompleto	Medium
	Bachillerato completo	Medium
	Técnico	Medium
	Tecnológico	Medium
	Licenciatura o pregrado	High
	Especialización	High
	Maestría o un nivel equivalente	High
	Doctorado o un nivel equivalente	High
	Otro	Excluded (set to NA)
**Libya**	Illiterate	Low
	Primary stage	Low
	Secondary school	Medium
	Tertiary school	Medium
	Bachelor’s degree or equivalent	High
	Master’s degree or equivalent	High
	Other	Excluded (set to NA)
**China**	Illiterate	Low
	Not completing primary school	Low
	Sishu/Old-style private school	Low
	Primary school	Low
	Middle school	Medium
	High school	Medium
	Vocational school	Medium
	Two-/Three-year college / Associate degree	High
	Four-year college / Bachelor’s degree	High
	Master’s degree	High
	Doctoral degree / Ph.D	High

## Abbreviations

CAPI: Computer-Assisted Personal Interviewing
CFA: Confirmatory factor analysis
CFI: Comparative Fit Index
LMM: Linear mixed-effects model
MGCFA: Multi-group confirmatory factor analysis
OECD: Organisation for Economic Co-operation and Development
SRMR: Standardized Root Mean Square Residual
TRAPD: Translation, Review, Adjudication, Pretesting, and Documentation
UMIC: Upper middle-income country
WHO: World Health Organization
